# T Cell Dysregulation in Rheumatoid Arthritis: from Genetic Susceptibility to Established Disease

**DOI:** 10.1007/s11926-025-01180-1

**Published:** 2025-01-25

**Authors:** Athena Chin, Annabelle Small, Soon Wei Wong, Mihir D. Wechalekar

**Affiliations:** 1 Department of Rheumatology, Flinders Medical Centre, Adelaide, SA Australia; 2 College of Medicine and Public Health, Flinders University, Adelaide, SA Australia

## Abstract

**Purpose of Review:**

Rheumatoid arthritis (RA) is a complex autoimmune disease characterized by chronic inflammation of the synovial tissue, where T cells play a central role in pathogenesis. Recent research has identified T peripheral helper (Tph) cells as critical mediators of local B cell activation in inflamed tissues. This review synthesizes the latest advancements in our understanding the of the role of T cells in RA, from initiation to established disease.

**Recent Findings:**

We explore recent advances regarding the genetic and epigenetic factors that predispose individuals to RA, the mechanisms of T cell activation and differentiation, and the interactions between T cells and other immune and stromal cells within the synovial microenvironment. The emergence of Tph cells as key drivers of RA pathobiology is highlighted, along with their potential as therapeutic targets. We also discuss the heterogeneity of T cell responses and their interplay with synovial cells, while addressing critical research gaps such as the drivers of T cell recruitment and the plasticity of synovial phenotypes.

**Summary:**

A deeper understanding of T cell dynamics in RA will provide valuable insights for developing targeted therapies to modulate T cell-mediated inflammation and improve patient outcomes.

## Introduction

Rheumatoid arthritis (RA) is a chronic autoimmune disorder that is characterized by inflammation of the specialized tissue that lines the joints (synovial tissue; ST). The pathogenesis of RA is complex, and involves a combination of genetic predisposition, environmental factors, and immune system dysregulation. ST analyses have revealed a central role of T cells in local inflammation, with a recent focus on a particular subset of PD-1^hi^ CXCR5^−^ CD4^+^ T cells, designated peripheral helper T (Tph) cells, which provide B cell help in inflamed tissue outside of germinal centres [1, 2]. In this review, we will synthesise the latest developments in our understanding of T cells in RA from early through to established disease. We discuss the underlying factors that contribute to RA, provide insight into current areas of research linking T cells with RA and critical research gaps, and highlight the recent groundbreaking findings that are reshaping concepts of RA pathobiology.

## The T Cell Response in RA: Genetic and Epigenetic Predispositions

**Fig. 1:**
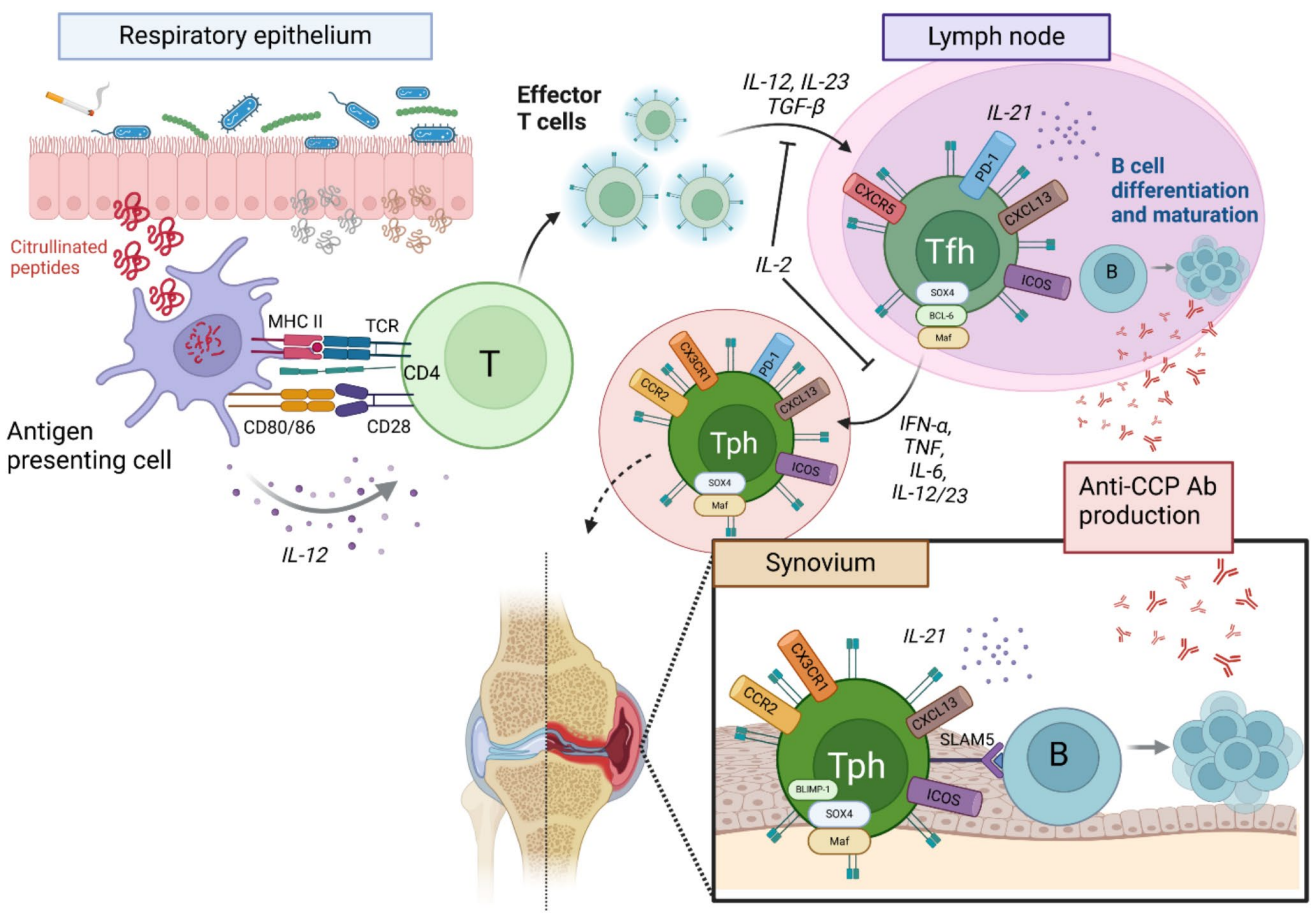
Tph Cell Differentiation and Function in RA. Citrullinated peptides originating from the respiratory epithelium trigger immune activation, with antigen-presenting cells (APCs) presenting peptides to naive T cells through MHC-TCR interaction. Differentiation into Tph cells is driven by cytokines (e.g., IL-12, IL-23, TGF-β), leading to the development of Tph (CXCR5^−^, PD-1^+^, CCR2^+^, CXCL13^+^). Tfh (CXCR5^+^) cells remain in lymph nodes to help B cell maturation, while Tph cells migrate to inflamed synovial tissue, promoting local B cell activation and anti-citrullinated protein antibody (anti-CCP Ab) production, further perpetuating joint inflammation.

T cell activation occurs in the context of MHC (Class I for CD8^+^ and Class II for CD4^+^) presentation of specific peptides to a cognate T cell receptor (TCR) alongside activating co-signals such as those provided through CD28 or CD40L (Fig. 1). Differentiation into helper subsets is further guided by cytokines. In genetically susceptible individuals, it is thought that environmental triggers modify self-antigens that are then presented by antigen-presenting cells (APCs) to self-reactive T cells, initiating pre-RA. The resultant, self-perpetuating T cell response is shaped by genetic and epigenetic factors, expanding to involve additional cell types as the disease progresses from pre-RA to established RA.

### Genetic Factors

The initiation of RA is primarily driven by genetic and epigenetic predispositions, and compelling evidence links these to the T cell response. Genome-wide association studies have identified over 100 RA risk loci, many linked to T cell regulation and immune function [3, 4]. Among these, the HLA-DRB1 “shared epitope” alleles are strongly associated with RA, promoting presentation of citrullinated peptides to CD4^+^ T cells that assist in generating anti-citrullinated peptide antibodies (ACPA), a key autoantibody in RA [4–6]. Recent work [4] that utilized expression quantitative trait locus (eQTL) analysis to explore the link between genetic variants and gene expression in RA, comparing ST and blood, identified a specific eQTL at HLA-DPB2 (rs3128921) that correlated with synovial expression, the lympho-myeloid pathotype, and more severe clinical disease. Other relevant genes such as *REL*, which affect NF-κB signalling pathways, and *STAT4*, involved in cytokine signalling, also contribute to T cell-mediated autoimmunity in RA [8, 9]. Finally, inhibitors of T cell activation are strongly associated with RA: polymorphisms in *CTLA4*, the ligand for CD28 and a negative regulator of T cell activation, lead to altered T cell responses and increased disease susceptibility [10], and a single nucleotide polymorphism in *PTPN22*, a tyrosine phosphatase expressed by haematopoietic cells that inhibits T cell signalling, is one of the most important non-HLA genetic risk factors for RA [11].

### Epigenetic Factors

Environmental factors, such as smoking, dietary habits, obesity, and overall lifestyle significantly contribute to RA pathogenesis. Smoking, particularly in individuals with HLA-DRB1 shared epitope alleles, increases the risk (25-fold) of developing RA by promoting citrullination of proteins that drive T cell autoreactivity [11–13]. Additionally, dysregulation of non-coding RNAs (microRNAs and long non-coding (lnc) RNAs) impacts T cell activity and inflammatory pathways, contributing to RA development [15]. Non-coding RNAs influence effector cell function by regulating NF-κB (miR-146a) [16], promote T helper (Th) 17 differentiation (miR-155) [17] and enhance Th1/Th17 responses (miR-21) [18]; lncRNAs can promote fibroblast proliferation (HOTAIR) [19, 20] and modulate inflammation (NEAT1) [21]. Altered DNA methylation and histone modifications can lead to the dysregulation of genes essential for immune tolerance, promoting autoimmunity. For instance, hypermethylation of the *CTLA4* promoter and hypomethylation of enhancer region upstream of *FOXP3* promoter reduces its respective expression, compromising regulatory T cell function (Cribbs et al., 2014; Kennedy et al., 2014). In addition, causal single nucleotide polymorphisms often map to T cell enhancers and transcription factor binding sites like IRF4, indicating that epigenetic mechanisms significantly influence T cell function in RA (Farh et al., 2015).

## T Cell Dynamics in Pre-RA and Early Disease

At mucosal surfaces, activated T cells help B cells to produce IgA antibodies as the first line of immune protection [22]. In seropositive RA, elevated ACPA and/or rheumatoid factor (RF) are a hallmark and are often detectable prior to development of inflammatory arthritis, indicating that the initial loss of immune tolerance may occur years prior to symptomatic disease [22–24]. The presence of IgA isotypes in RA therefore suggests that this may first occur at mucosal sites (such as the lungs or gut), with local loss of antigen tolerance in high-risk individuals progressing to a systemic response and later migrating to the synovial joints [26] (Fig. 1). The lungs particularly have been implicated as a site for immune exposure to citrullinated self-peptides due to citrullination-inciting factors such as smoking, and distinct studies with bronchoalveolar lavage (BAL) of RA patients have separately demonstrated the presence of B cells sensitised to citrullinated proteins [27], in addition to ACPAs in BAL fluid [28].

Various T cell subsets have been associated with the early stages of RA and antibody production. Recently, in a study of pre-RA, significant expansions of T cell subsets were observed including CCR2^+^CD4^+^ T cells, Tph, Th1, and CXCR5^+^CD8^+^ T cells, indicating the importance of T cells prior to establishment of disease [29]. Here, the role of these will be further discussed.

### CD4+ T Cells

To mount a normal immune response, CD4^+^ T cell differentiation is controlled by cytokines and transcription factors, capable of producing subtypes of Th1, Th2, and Th17 cells, or T follicular helper cells (Tfh) depending on initial insult. The transcription factor T-bet (*TBX21*) drives interleukin (IL)-12 and interferon (IFN)-γ production, resulting in Th1 cells that secrete IFN-γ; GATA3 drives production of IL-4, which in turn promotes Th2 differentiation and subsequent secretion of IL-4, IL-5 and IL-13. Meanwhile, RORγt drives IL-6, IL-23 and IL-1β-mediated Th17 differentiation, which in turn produce IL-17 [30].

In early and established RA, the proportions of CD4^+^ T cells in the ST relative to blood are elevated [31, 32], and an imbalance between Th1 and Th2 cells drives a proinflammatory state. Increased IFN-γ, TNF and IL-2 drives local cartilage destruction and bone erosion [33], while Th2 cytokines (IL-4, IL-13) which usually exert an inhibitory effect on osteoclasts and bone resorption [34]. Th17 cells also play an important role in the pathogenesis of RA; IL-17 contributes to proinflammatory cytokine production and works synergistically with IL-1 and TNF to cause bone erosions secondary to increased prostaglandin E2 [35].

### CD8+

The association of MHC class I alleles with RA suggests a role of CD8^+^ T cells in driving disease [36]. Indeed, cytotoxic CD8^+^ T cell populations are distinct in RA compared to healthy individuals, with greater representation of CD27^−^CD62L^−^ subsets that produce more granzymes and pro-inflammatory cytokines [37]. In ACPA positive patients, CD8^+^ T cells specific for citrullinated antigens have been identified with clonal expansion of CD8 + cells expressing granzymes (GZMK, GZMB) that appear to be specific to synovial tissue of RA patients [38]. In addition, a recent report [29] of an expanded CXCR5^+^CD8^+^ T cell population expressing multiple chemokine receptors (CXCR3, CX3CR1, and CCR4) in the blood of at-risk individuals suggests that CD8^+^ cells are important prior to establishment of disease. Finally, with a combination of single cell and functional approaches, Moon, et al. (2023) [39] identified expanded *GZMB*^+^ and *GZMK*^+^*CD8*^+^ T cell populations expressing cytotoxic, pro-inflammatory and tissue homing transcriptional programs in the blood of seropositive RA patients. They demonstrated expanded *GZMB*^+^ populations highly expressing *GNLY* that were present in the RA ST, and stimulation of ACPA + RA CD8^+^ T cells with citrullinated antigens in the context of HLA class I induced cell proliferation, clonal expansion, and expression of cytotoxic mediators and chemokine receptors, demonstrating that CD8^+^ cells targeting citrullinated proteins may contribute to synovitis and joint tissue destruction [39].

### Memory T Cells (Resident Memory)

Following an immune response, subsets of effector T cells undergo transition to become memory T cells, providing a front-line of defence against re-infection [40]. These consist of three major subtypes: central memory (Tcm) (CD62L^+^CCR7^+^), effector memory (Tem) (CD62L^−^CCR7^−^), and resident memory (Trm) [41, 42]. While Tcm and Tem circulate through the bloodstream and lymph system, Trm take up residency in non-lymphoid tissue and can persist long after formation [43]. Synovial fluid T cells expressing Trm markers (CD8^+^CD69^+^ CD103^+^) in RA were first identified in 2020 [44]. Recent characterisation of Trm in the RA ST by gene expression profiling and surface marker analysis revealed that CD8^+^ Trm cells (CD69^+^CD103^+^) were more prevalent than CD4^+^ [45]. Microarray data further demonstrated the presence of Trm signatures in lymphoid RA ST [45]. Additionally, in murine models, cells bearing a comparable signature were observed to persist in previously inflamed joints following resolution of inflammation [45]. Taken together, these suggest the future potential for targeting Trm populations in established RA.

### Treg

Treg memory subsets are essential for maintaining immune tolerance and preventing autoimmunity by secreting anti-inflammatory cytokines, including IL-10, TGF-β, and IL-35 and to inhibit effector T cell function [46]. In RA, these Treg memory populations exhibit impaired functionality, contributing to the chronic inflammatory state [47]. Additionally, the signalling through TNF receptors plays a significant role in modulating Treg function. TNF receptor Type I (TNFR1) and Type II (TNFR2) have opposing effects on Tregs: engagement of TNFR1 tends to inhibit Treg suppressive capabilities and promotes inflammatory pathways, thereby perpetuating immune activation. In contrast, activation of TNFR2 enhances Treg stability and their immunosuppressive functions, facilitating the resolution of inflammation [48]. This differential signalling through TNF receptors exacerbates the dysfunctional Treg response in RA, highlighting potential therapeutic targets aimed at restoring Treg functionality and achieving immune homeostasis. Understanding the balance between TNFR1 and TNFR2 signalling in Treg subsets offers promising avenues for mitigating the autoimmune processes underlying RA.

### T Follicular and T Peripheral Helper Cells

In the circulation, IgG subclasses of ACPA are the most common [49]. These are produced following naïve B cell differentiation, class switching, and maturation into specialised IgG-producing plasma cells. The key effector Tfh cells express the chemokine receptor CXCR5, which facilitates movement from the circulation into lymph node germinal centres where they provide help to B cells, facilitating this differentiation. This is through cell-contact-dependent stimulation via CD40L and ICOS from Tfh cells, interacting with CD40 and ICOSL on B cells respectively, in conjunction with signals provided by IL-21 (Fig. 1).

In RA, help to B cells can be provided locally in the ST, however Rao, et al. (2017) demonstrated that Tph rather than Tfh are responsible and are predominant over Tfh [2, 31, 50]. Without CXCR5, and with the expression of the chemokine receptors CCR2 and CX3CR1, Tph are functionally similar to Tfh, and can migrate through the peripheral blood and into the inflamed non-lymphoid tissue, where they assist in B cell maturation and perpetuate adaptive immune system activation in peripheral organs [50]. Tph are highly represented in the ST with suggestion of active proliferation, and clonally expanded cells appear to have upregulation of effector and activation signatures, reflecting persistent local B cell stimulating function [32]. This is consistent with Rao et al. [2] demonstrating the local pathogenic role of Tph cells in RA, where synovial studies showed expansion of PD-1^+^CXCR5^+^CD4^+^ T cells inducing greater differentiation of memory B cells into plasma cells, via IL-21 and signalling lymphocytic activation molecule-5 (SLAM5).

Tph are further distinguished from Tfh by lower *BCL6* levels and higher *PRDM1* (BLIMP-1) [1]. Single cell ATAC-seq (assay for transposase-accessible chromatin with sequencing) further revealed that Tph in RA also have a distinct chromatin landscape enriched for BATF/AP-1 transcription factor motifs [51]. This BATF/AP-1 complex drives pro-inflammatory gene expression, stabilizes Tph cell identity, and enhances chromatin accessibility, contributing to the ability of Tph cells to support B cell activity and pathogenesis [51]. In vitro, Tfh-like CD4^+^ T cells (PD-1^+^CXCR5^+^) can be induced by culture in the presence of anti-CD3 and -CD28 antibodies, along with IL-12 and TGF-β; however with the further addition of IFN-α, these cells better resemble T peripheral helper cells (Tph) (PD-1^+^ CXCR5^−^) [52].

Overabundant immune T cell phenotypes have been observed in the blood of individuals at-risk of RA due to family history and/or ACPA positivity, with mononuclear cell (PBMC) analysis demonstrating expanded Tph cell populations in addition to CCR2^+^CD4^+^ T cells [29, 53]. Further analysis of these cells showed Th17 phenotypical effector function, with production of cytokines implicated in the pathogenesis of early RA, such as IL-17 and IFN-γ [49], demonstrating the role of Tph in RA locally and systemically in pre- and early RA.

### Mucosal-Associated Invariant T (MAIT) Cells

Mucosal-associated invariant T (MAIT) cells are a subset of innate-like T cells that recognize microbial metabolites presented by the MHC class I-related protein, MR1. These cells, enriched in mucosal locations, produce inflammatory cytokines such as IL-17, TNF, and IFN-γ and have been implicated in the pathogenesis of RA, particularly within the ST. A recent single-cell study [32] identified shared clones of MAIT cells between blood and synovium, along with clones uniquely expanded in the synovium. Within the RA ST, MAIT cells exhibit phenotypic and functional alterations, including an increased proportion of CD4^+^ MAIT subsets, reduced CD161 expression, and hyporesponsiveness to bacterial stimulation [50]. These changes may impair the immune system’s ability to regulate microbial components in the joint, thereby contributing to synovial inflammation. Collectively, these findings suggest that MAIT cells may act as a critical link between microbial dysbiosis and autoimmune progression in RA, highlighting their potential as a therapeutic target in synovial inflammation [51].

## Immune Aging in Established RA

Accelerated T cell aging contributes to persistent inflammation in established RA as a result of earlier exhaustion and involution of the thymus. This results in a loss of TCR repertoire and compensatory peripheral T cell expansion of CD28^−^ T cells [52]. CD28 is pivotal for T cell proliferation, and this population of prematurely immunosenescent CD28^−^ cells include CD45RA^+^ memory T cells that produce inflammatory cytokines (IFN-γ, TNF, IL-1β and IL-6) [53]. CD28^−^ cells also appear to acquire NK cell receptors and granzyme production, and it is postulated that the reduction in natural T and NK cell production favours restitutive expansion and development of such cellular phenotypes [54], which subsequently promotes a tenacious inflammatory state in RA.

## Metabolic Dysfunction in T Cells

Changes in T cells on a molecular level are pivotal in driving persistent inflammation in established RA due to development of mitochondrial defects with metabolic dysfunction [55]. Yang et al. [56] demonstrated that glucose metabolism in CD4^+^ RA T cells is altered, with shunting of normal pathways towards the pentose-phosphate pathway (PPP) due to suppression of glycolytic enzyme 6-phosphofructo-2-kinase (PFKFB3). This results in reduced pyruvate and increased lactate; an acidic state that favours T cell differentiation into Th17 cells [57]. Furthermore, PPP upregulation increases nicotinamide adenine dinucleotide phosphate (NADPH) and reduces overall reactive oxygen species in these T cells, which inhibits activation of a cell cycle kinase ataxia telangiectasia mutated (ATM) [58]. RA T cells are thereby able to bypass certain cell cycle checkpoints and undergo accelerated proliferation and differentiation to Th1, Th17 and memory cells, resulting in a pervasive pro-inflammatory state in patients with established RA [59]. The altered cellular metabolism in RA T cells also appears to directly contribute to mobilisation into synovium. Shen et al. [60] observed this low pyruvate, high NADPH state upregulates podosome scaffolding protein (TKS5); T cells with high expression of this protein spontaneously formed membrane ruffles, a process essential for cell motility and migration to non-lymphoid tissue. As such, alterations in T cell metabolism drives both migration into synovium and accelerated differentiation, resulting in a recalcitrant pro-inflammatory environment at a local level.

## T Cell Interactions in RA

Interactions between T cells and synovial fibroblasts were demonstrated in vitro binding assays in 1988 [61]. Since then, the advances in single-cell and spatial technologies have significantly advanced our understanding of stromal cells and their role in local inflammation. Two FAPα^+^ fibroblast populations were initially described in the RA ST: THY1^+^ fibroblasts sublining-fibroblasts, which drive more severe and persistent inflammatory arthritis, and THY1^−^ lining layer fibroblasts, which mediate bone and cartilage damage [62]. Since the documentation of these populations, THY1^+^ and THY1^−^ fibroblasts were demonstrated to exist in a transcriptional gradient, controlled by endothelial cells and NOTCH3 signalling [68]. While THY1^−^ fibroblasts had little effect on inflammation [62], in mice with collagen-induced arthritis (CIA), injection of FAPα^+^THY1^+^ fibroblasts resulted in increased effector CD4^+^ T cells and reduced FOXP3^+^ T regulatory cells, demonstrating these possess an ‘immune effector’-like phenotype, capable of directly modifying the immune millieu.

Macrophages are abundant within the RA ST and positively correlate with disease activity [63, 64]. As sentinel cells, macrophages have critical roles in the initiation and resolution of the inflammatory response, tissue repair, and the maintenance of homeostasis [65]. While limited studies have investigated the interactions between Tph and macrophages in the RA ST, IL-21 is able to induce CXCL8 production by macrophages. In synovial fluid, this has an anti-inflammatory effect, reducing production of TNF, IL-6, IL-8, IL-1β, and IL-12 [66].

Increased understanding of the pro-inflammatory and homeostatic interactions occurring between local cells in the ST will be pivotal to the development of therapeutic approaches targeting these. Newer technologies combining omics technologies while retaining spatial information will be key to these advancements.

## Personal Observations and Controversial Hypotheses

### Innovative Findings from Current Research

**Fig. 2:**
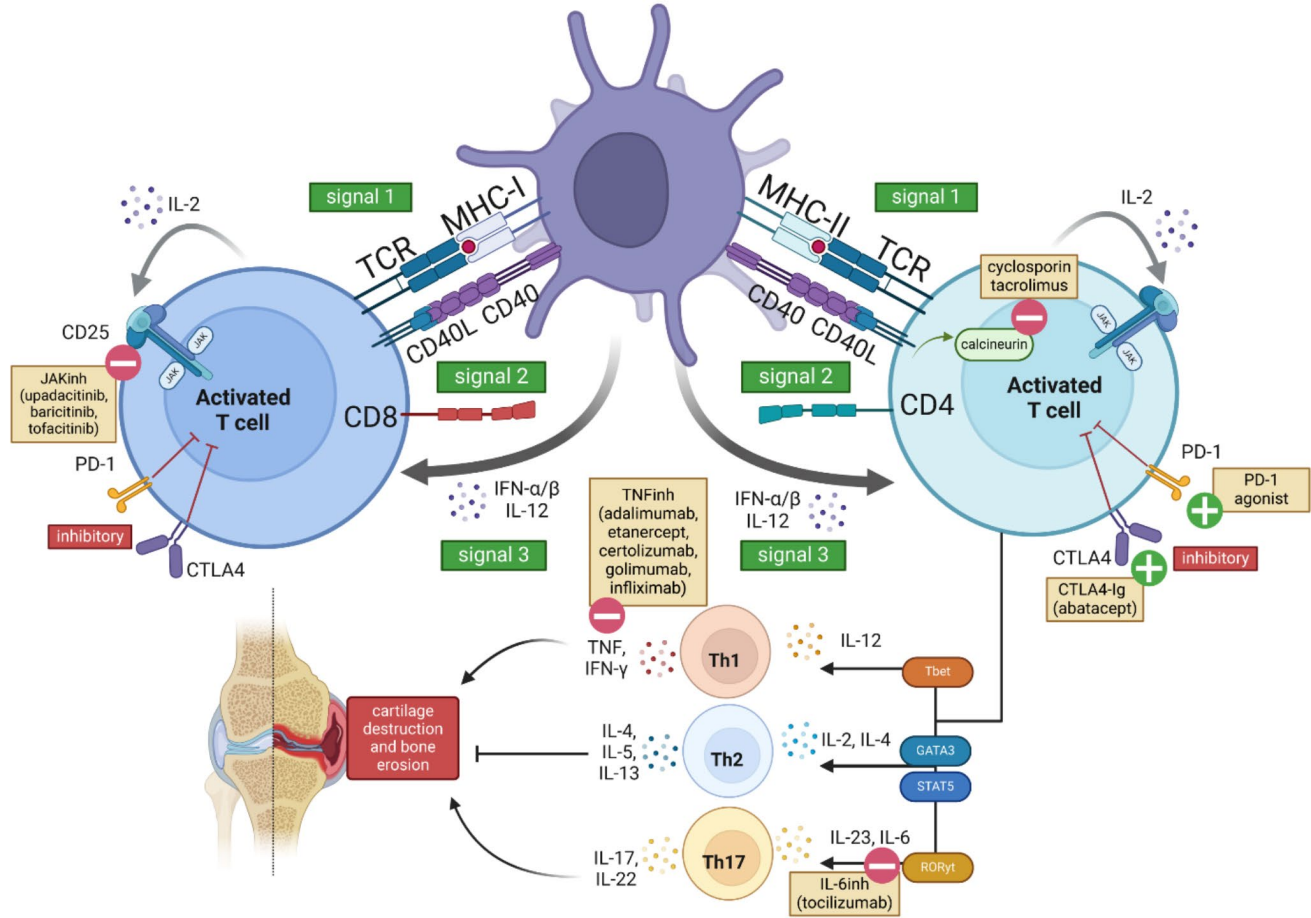
Overview of T Cell Activation and Therapeutic Targets in RA. The activation of CD4^+^ and CD8^+^ T cells by antigen-presenting cells (APCs), highlighting three key signals: MHC-TCR interaction (Signal 1), co-stimulatory molecules (Signal 2), and cytokine-driven differentiation (Signal 3). The roles of therapeutic agents are shown, including JAK inhibitors, TNF inhibitors, CTLA4-Ig (abatacept), calcineurin inhibitors, IL-6 inhibitors, and PD-1 modulation in inhibiting T cell activation, differentiation, and inflammatory response. The differentiation of helper T cells (Th1, Th2, Th17) and their effects, such as cartilage destruction and bone erosion, are also depicted.

Our recent investigation revealed the presence and expansion of Tph in the peripheral blood and ST of early, ACPA^+^, treatment-naive RA (< 12 months of symptom onset and fulfilling the 2010 American College of Rheumatology/EULAR classification criteria) compared to the healthy blood and osteoarthritis ST respectively [31]. As there is a recognised window of opportunity for the optimal treatment response in RA [67], the finding of these within the tissue in early disease suggests that modulating Tph activity may be a possible early therapeutic avenue. High expression of the inhibitory checkpoint molecule PD-1 on these cells may therefore be an effective target for agonistic approaches (Fig. 2); indeed, in a recent phase 2a trial using Peresolimab, a humanised anti-PD-1 monoclonal antibody have demonstrated therapeutic efficacy in the form of a significant reduction in DAS28-CRP by week 12 [68]. Additionally, our group demonstrated not only that PD-1 expression is high the RA synovium, but its ligand, PD-L1, is conspicuously absent [69]. Thus, to investigate whether PD-L1 expression could be restored, our preliminary study demonstrated that the commonly used therapeutics in RA, methotrexate and anti-TNF, modulate PD-L1 expression on isolated early RA synovial macrophages, suggesting that modulation of the PD-1 pathway may be a mechanism of action of these therapeutics in early disease [76].

A current intriguing area of RA research is that of ST phenotypes, first described by Dennis et al. [70] as ‘myeloid’, ‘lymphoid’, and ‘low-inflammatory’, and ‘fibroid’. Bulk RNA-sequencing refined these into the fibroblastic ‘pauci-immune’ pathotype, the macrophage-rich ‘diffuse-myeloid’ pathotype, and a ‘lympho-myeloid’ pathotype characterized by infiltration of lymphocytes and myeloid cells [71]. More recently, single-cell sequencing has allowed the further characterised into ‘cell-type abundance phenotypes’, or ‘CTAPs’, defined by cell composition [79]. These studies have all revealed some association of synovial phenotype with clinical outcome [77–79], however important critical questions remain. The R4RA trial highlighted by Zhang et al. [72] illustrates that ST CTAPs are not static; they change in response to treatment. Notably, our own unpublished data from our early, ACPA positive, inception RA cohort study’s bulk RNAseq CTAP prediction analyses further support this dynamic shift in pathotypes post-treatment. This raises compelling questions: Do these changes reflect a therapeutic effect, or might they indicate a continuum of disease progression? Is tissue phenotype the same from different joints within the same patient? Can synovial phenotype prior to treatment initiation predict treatment response and patient prognosis? Further in-depth ST studies, facilitated by the development of spatial analyses on archival tissue samples, will be required to answer these pressing clinical questions.

### Controversial Areas in Current Research

One of the more controversial areas in RA research involves the heterogeneity of T cell responses across different patients and even within the same patient over time. Notably, T cells can exhibit both pathogenic and protective roles, which adds complexity to understanding their contributions to RA disease progression. Recent single-cell RNA sequencing studies, including work by Zhang et al. [72] have highlighted this heterogeneity by identifying multiple subsets of CD4⁺ and CD8⁺ T cells with distinct gene expression profiles within the RA synovium.

The duality of T cell roles is particularly evident in pauci-immune, myeloid-lymphoid interactions. Here, the same molecular signals can produce opposing effects, influenced by both the microenvironment and the stage of the disease. These complexities in T cell roles also translate into challenges for therapeutic targeting. While agents such as abatacept (CTLA4-Ig), which target T cell co-stimulation, have shown efficacy, therapies aimed at specific T cell cytokines, like IL-17 inhibitors, have produced mixed results. Despite promising findings from murine studies and human in situ data, clinical trials with IL-17 inhibitors, such as secukinumab and brodalumab, have not consistently shown benefit in RA patients [73–77]. This inconsistency raises questions about the pathogenic versus protective roles of certain T cell subsets and cytokines in different patient populations, and suggests the importance of IL-17 in disease may be specific to a certain stage or subset of RA.

Furthermore, the microenvironment plays a pivotal role in shaping T cell function. In RA, the ST and synovial fluid are rich in cytokines and chemokines that profoundly influence T cell behaviour. For instance, the presence of IL-15 in the synovium can promote the survival and activation of pathogenic T cells [78]. Interactions with other immune cells, such as dendritic cells and macrophages, also modulate T cell responses. These complex interactions further contribute to the heterogeneity observed in RA.

## Summary and Future Directions

RA presents with remarkable heterogeneity and diverse outcomes, but despite this, T cells are a common central player in the autoimmune reaction underlying the disease, highlighting the critical need to comprehensively understand their dynamic and diverse roles. Future research should therefore focus on elucidating the mechanisms by which T cells interact with other cells across different synovial environments and disease stages, how these relate to treatment response and prognosis, and how the intricate immunological mechanisms that control T cell activation are altered in a way that allows disease to progress and persist. Insights into these pressing questions could lead to a more holistic view of RA pathogenesis and have the potential to inform the development of T cell-specific therapies at different stages of disease.

Considerable gaps remain in our understanding of the precise triggers that initiate RA and T cell-mediated pathology, the reasons for variability in T cell responses among patients, and the potential for targeting early T cell activation states to prevent disease progression. Furthermore, questions such as how T cells are recruited into the synovium in RA remain only partially answered. Is it citrullinated epitopes of specific proteins, and if so, where are these epitopes most influential? These areas of uncertainty present valuable directions for future research.

Finally, the insights to be gained from studying T cell dynamics will have profound implications for clinical practice. Identifying biomarkers of T cell activity could enhance early diagnosis and allow for more precise targeting of therapies, meeting what is a current pressing need for clinicians. Additionally, understanding the specific roles of T cell subsets in RA could lead to the development of novel therapeutic agents that selectively modulate T cell functions, reducing side effects and improving efficacy.

## Conclusions

This review highlights the complex and pivotal role of T cells and the emerging Tph subset in the pathogenesis and progression of RA. By integrating the latest approaches in genetic, epigenetic, tissue, and cellular analyses, we will gain a richer understanding of T cells and RA, which will together inform more targeted and effective therapeutic strategies for patients living with RA.

## Key References

•**Goldmann K**,** Spiliopoulou A**,** Iakovliev A**,** Plant D**,** Nair N**,** Cubuk C**,** et al. Expression quantitative trait loci analysis in rheumatoid arthritis identifies tissue specific variants associated with severity and outcome. Ann Rheum Dis. 2024;83(3):288 − 99. doi**: 10.1136/ard-2023-224540.

This study used eQTL analysis to explore the link between genetic variants and gene expression in RA ST and blood. This revealed a specific eQTL at HLA-DPB2 (rs3128921) that correlates with synovial expression, clinical severity, and the lympho-myeloid pathotype.

•**Inamo J**,** Keegan J**,** Griffith A**,** Ghosh T**,** Horisberger A**,** Howard K**,** et al. Deep immunophenotyping reveals circulating activated lymphocytes in individuals at risk for rheumatoid arthritis. bioRxiv. 2023. doi**: 10.1101/2023.07.03.547507.

In this pre-print, mass cytometry was applied to deeply characterize the immunophenotypes in blood from individuals at risk of RA, judged by ACPA status and first-degree relatives. Significant cell expansions were observed in at risk individuals, and an RA immunophenotype score capable of distinguishing at-risk from healthy was developed.

•**Moon JS**,** Younis S**,** Ramadoss NS**,** Iyer R**,** Sheth K**,** Sharpe O**,** et al. Cytotoxic CD8(+) T cells target citrullinated antigens in rheumatoid arthritis. Nat Commun. 2023;14(1):319. doi**: 10.1038/s41467-022-35264-8.

This work used single cell RNA and TCR sequencing of ACPA^+^ RA patient CD8^+^ T cells and identified *GZMB*^+^ subpopulations containing large clonal lineage expansions. Citrullinated autoantigens presented by MHC class I were shown to activate and expand RA blood-derived *GZMB*^+^*CD8*^+^ T cells, inducing expression of cytotoxic mediators and killing of target cells.

•**Weinand K**,** Sakaue S**,** Nathan A**,** Jonsson AH**,** Zhang F**,** Watts GFM**,** et al. The chromatin landscape of pathogenic transcriptional cell states in rheumatoid arthritis. Nat Commun. 2024;15(1):4650. doi**: 10.1038/s41467-024-48620-7.

This work comprehensively examined genome-wide open chromatin at the single cell level in the RA ST. A total of 24 chromatin classes were identified, and an RA chromatin ‘atlas’ was generated. Specifically, this showed that Tph have a distinct chromatin landscape enriched for BATF/AP-1 transcription factor motifs that drive pro-inflammatory gene expression, stabilizes cell identity, and enhances chromatin accessibility.

•**Wei K**,** Korsunsky I**,** Marshall JL**,** Gao A**,** Watts GFM**,** Major T**,** et al. Notch signalling drives synovial fibroblast identity and arthritis pathology. Nature. 2020;582(7811):259 − 64. doi**: 10.1038/s41586-020-2222-z.

Using transcriptome and organoid studies, Wei and colleagues show that NOTCH3 and Notch target genes are upregulated in RA synovial fibroblasts and that NOTCH3 signalling drives transcriptional and spatial gradients. Their work indicates that synovial fibroblasts exhibit a positional identity regulated by Notch signalling, underlying inflammation and pathology in RA.

•**Tuttle J**,** Drescher E**,** Simón-Campos JA**,** Emery P**,** Greenwald M**,** Kivitz A**,** et al. A Phase 2 Trial of Peresolimab for Adults with Rheumatoid Arthritis. N Engl J Med. 2023;388(20):1853-62. doi**: 10.1056/NEJMoa2209856.

This trial demonstrates early evidence of the efficacy of agonistic PD-1 therapy for patients with moderate to severe RA, implicating dysregulation of the PD-1 axis in RA pathogenesis.

•**Zhang F**,** Jonsson AH**,** Nathan A**,** Millard N**,** Curtis M**,** Xiao Q**,** et al. Deconstruction of rheumatoid arthritis synovium defines inflammatory subtypes. Nature. 2023;623(7987):616 − 24. doi**: 10.1038/s41586-023-06708-y.

In this work, dissociated synovial cells were profiled using multi-modal single-cell RNA-sequencing and surface protein data coupled with ST histology. Tissues were characterised as CTAPs characterized by selectively enriched cell states, which are dynamic and capable of predicting therapeutic response, demonstrating the striking diversity of the RA ST between patients.

## Data Availability

No datasets were generated or analysed during the current study.
